# Establishing and maintaining the National Vaccination Register in Finland

**DOI:** 10.2807/1560-7917.ES.2017.22.17.30520

**Published:** 2017-04-27

**Authors:** Ulrike Baum, Jonas Sundman, Susanna Jääskeläinen, Hanna Nohynek, Taneli Puumalainen, Jukka Jokinen

**Affiliations:** 1 Department of Public Health Solutions, National Institute for Health and Welfare, Helsinki, Finland; 2 Department of Health Security, National Institute for Health and Welfare, Helsinki, Finland

**Keywords:** vaccines and immunisation, vaccination register, primary health care, patient information systems, vaccine lot number, Finland, immunisation information systems, vaccination coverage, vaccination records

## Abstract

Computerised, population-based vaccination registers are valuable tools for assessing the vaccine uptake and impact in populations. However, reliable impact assessment is only possible if the data quality can be reviewed and monitored continuously. This report describes the establishment and maintenance of the National Vaccination Register (NVR) in Finland. Currently, the NVR covers nationwide records of vaccinations given within the frame of the National Vaccination Programme since 2009. All vaccinations registered in the NVR contain a record of the personal identity code, the administered vaccine, and the date of vaccination. The vaccine lot number is the key component for recording and identifying vaccinations, because of its broad availability across patient information systems and its importance in vaccine safety monitoring. Vaccination records are accumulated and updated daily into the NVR, and their completeness is monitored monthly to assess deficiencies in data entry and data collection. Additionally, an alert system reports unexpected changes in data accumulation prompting the validation of observed changes in vaccination coverage. The presented process documentation may serve as basis to improve the design and quality of other vaccination or healthcare registers and aims to inspire the set-up of vaccination registers in those countries which still do not have one.

## Introduction

Computerised, population-based vaccination registers are valuable tools for assessing the vaccine uptake in populations in real-time. The performance of dynamic vaccination programmes can be evaluated, time trends monitored, and sub-populations with low vaccination coverage identified. Most importantly, by linking individual-level vaccination records with other medical records and health outcome databases, the impact of vaccines – both effectiveness and safety – can be studied comprehensively. This in turn will aid in formulating the best possible vaccination programmes.

**Figure 1:**
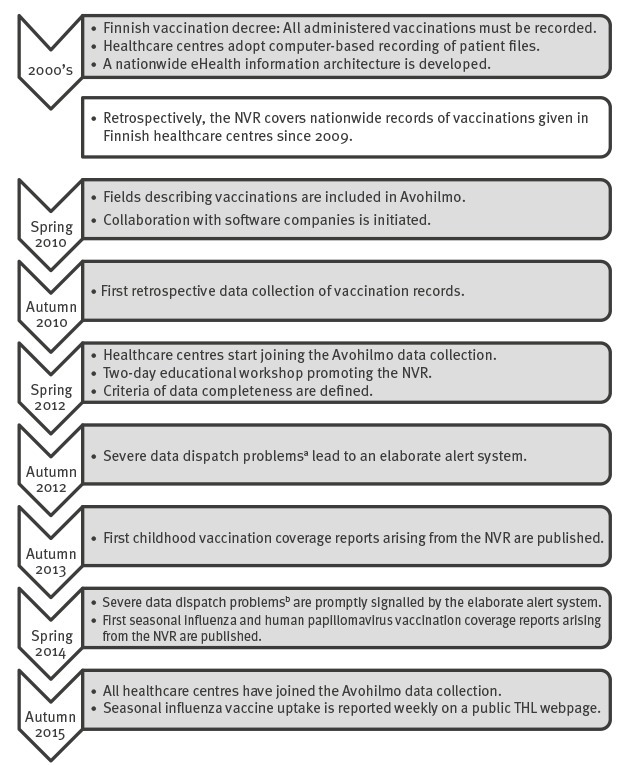
Milestones in the process of developing and maintaining the National Vaccination Register in Finland

The need for a National Vaccination Register (NVR) was recognised in Finland already in the late 1990´s and the Finnish vaccination decree of 2004 [1] requires all administered vaccinations to be recorded (Figure 1). In 2009, Finland introduced its NVR. Already before that, several countries in Europe had established vaccination registers on a regional and national level, e.g. Norway in 1995 [2], Denmark in 2000 [3], and the Netherlands in 2005 [4].

Avohilmo: Register of Primary Health Care Visits; NVR: National Vaccination Register; THL: National Institute for Health and Welfare.

^a^ After an update to one patient information system software, all vaccination records of newly and recently born children were accidentally omitted from real-time data submissions in the healthcare centres using that particular software.

^b^ After an update to one patient information system software, all adolescent human papillomavirus vaccinations were accidentally omitted from real-time data submissions in the healthcare centres using that particular software.

This report describes how the National Institute for Health and Welfare (THL) established the NVR in Finland. The presented process documentation may serve as a basis to improve the design and the quality of other vaccination or healthcare registers. Moreover, the aim is to inspire the set-up of vaccination registers in those countries which still do not have one.

## Structure of public primary healthcare delivery in Finland

**Figure 2:**
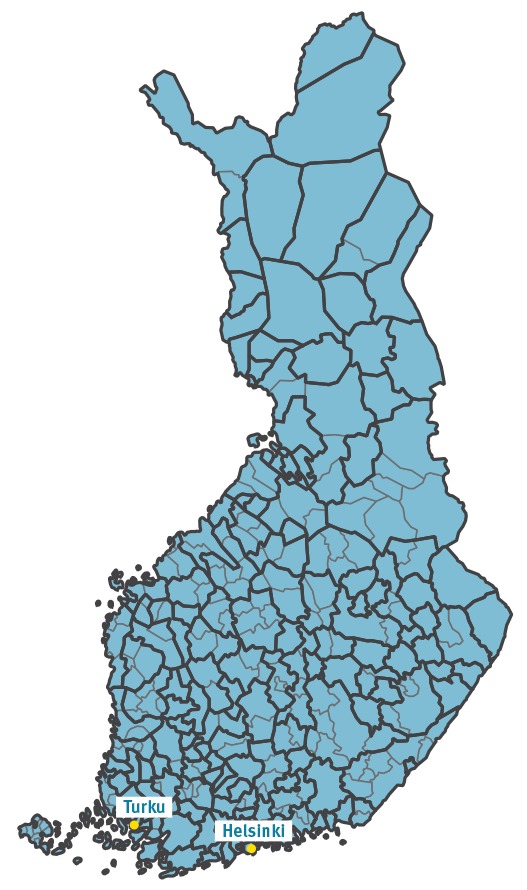
Map of administrative areas, municipalities and healthcare centres, Finland, 2015 (n=317 municipalities and 153 healthcare centres)

Finland has a population of 5.5 million and an annual birth cohort of ca 55,000 [5]. All vaccinations within the National Vaccination Programme (NVP) [6] are purchased centrally and paid by the state. They are given free of charge and on a voluntary basis. Municipalities (local governments) are responsible for the primary healthcare of their citizens, including NVP vaccinations. Changes in municipality borders and municipality mergers have occurred continuously in Finland during the last two decades. At the end of 2015, the population size of municipalities ranged from 99 in Sottunga, an island municipality of Åland, to 628,208 in the capital Helsinki [5]. In order to rationalise the organisation of public primary healthcare, some small municipalities have joined forces to form shared healthcare centres (HCCs), while other municipalities maintain their own. A map of Finnish administrative areas (317 municipalities and 153 healthcare centres in 2015) is presented in Figure 2.

Municipality borders are depicted in grey and healthcare centre boundaries are superimposed in black.

Finnish HCCs have adopted computer-based recording of patient files since the early 2000s. In the mid-2000s, nearly all HCCs were using electronic patient information systems (Figure 1). However, both the versions of the systems, and the systems themselves varied between HCCs. Today, five commercial software programmes are in use, but the commercial ownership of the software programmes has changed frequently.

## Standardisation and coding

As part of the nationwide eHealth information architecture [7] developed in the mid-2000s, a nationwide service for classification, coding, and terminology of health information was established. The providers of the patient information systems’ software generally apply the respective classifications, codes, and terminologies when implementing structured fields in their systems. In spring 2010, the nationwide coding of vaccination information was added to the National Code Server (Figure 1), where it is publicly available as reference [8]. It currently covers service providers, vaccines’ trade and generic names, vaccine preventable diseases, vaccination route, and vaccination site. Vaccines also appear in the Finnish Medicinal Products Database [9], which additionally covers the Anatomical Therapeutic Chemical code [10] and the Nordic Article Number [11].

### Vaccine lot number

**Figure 3:**
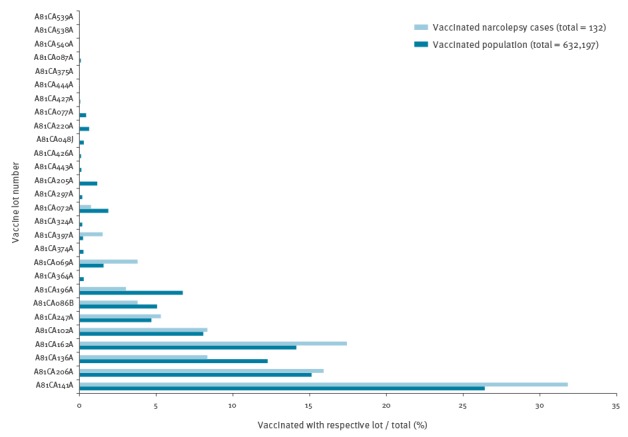
Pandemic influenza vaccine lot distribution in 4–19-year-olds who developed narcolepsy^a^ after vaccination compared with the corresponding population of vaccinated 4–19-year-olds, Finland, 2009–2012

^a^ Confirmed cases diagnosed by the end of 2012 [14].

Both the Finnish and the European Medicines Agency mandate a lot-level traceability for purposes of vaccine safety monitoring [12]. The importance of being able to trace vaccinations on a lot level was highlighted in a vaccine safety study conducted by THL, regarding the pandemic influenza vaccine and narcolepsy, a rare sleep disorder, in children and adolescents [13,14] (Figure 3), where one of the questions posed was whether the onset of narcolepsy was due to manufacturing error confined to certain lots of the vaccine.

The distribution of vaccine lots in those who developed narcolepsy does not differ from the distribution in the general population (chi-squared test for independence: p value = 0.315).

In this study, nationwide vaccination data collected from the HCCs’ patient information systems were linked to information from patient files collected from Finnish hospitals and reviewed by sleep disorder experts. Certain lot numbers occurred more frequently in patients who had developed narcolepsy (Figure 3). However, when comparing the relative frequency of lot numbers to the corresponding population, the lot number distributions did not differ, suggesting that the occurrence of the disease was not associated with certain lot numbers.

Based on these experiences, the vaccine lot number was recognised as the key content for identifying vaccinations in the NVR.

## Data entry and data collection

**Figure 4:**
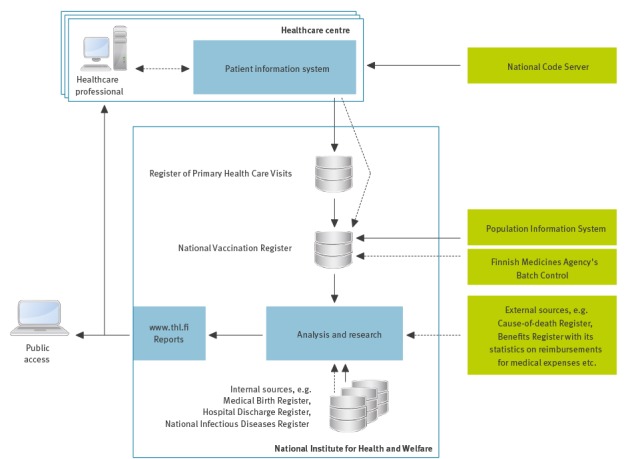
General architecture of the National Vaccination Register in Finland

The implementation of the NVR was guided by two principles: (i) to avoid double entry of data and (ii) to avoid double collection of data. The vaccination records in the NVR were therefore designed to be collected directly from the patient information systems and as part of other pre-existing nationwide register collections (Figure 4).

Automated processes are indicated by solid arrows and manual processes by dashed arrows.

In the late 2000s, in order to expand the accessibility of nationwide health-related information, THL initiated a project to collect nationwide information about primary healthcare visits and subsequently established the Register of Primary Health Care Visits (Avohilmo) as part of its statutory duties [15]. The first pilot to collect real-time data of primary healthcare visits was conducted in 2009. The fields describing vaccinations were included to the Avohilmo data content in spring 2010. This formed a basis for collecting the records of vaccinations given during any primary healthcare visit in real-time. The HCCs, which are responsible for the administration and computer-based recording of NVP vaccinations, joined the Avohilmo data collection gradually: the majority of HCCs (105/150) started submitting real-time data in 2012 and by autumn 2015 all operational HCCs (153/153) had joined Avohilmo (Figure 1).

The definition of Avohilmo’s data content has been evolving over time and all vaccinations given within the public primary healthcare system are covered. Table 1 shows the 2017 vaccination data content in Avohilmo.

**Table 1:** Variables in Avohilmo, the Register of Primary Health Care Visits, that are incorporated into the National Vaccination Register, Finland, 2017

Field	Coding of content	Currently used to identify
Client's personal identity number	Personal identity code	Vaccinee
Service provider	National health service provider code	Healthcare centre
Date and time of contact	Timestamp with minute precision	Date of vaccination
Vaccine administration date^a^	Timestamp with minute precision	NU
Lot number of vaccine	Free text	Administered vaccine
Trade name of vaccine	THL vaccine trade names code^b^	Administered vaccine
Generic name of vaccine^a^	THL vaccine generic names code^b^	NU
Vaccine preventable disease^a^	THL vaccine preventable disease code^b^	NU
Anatomical Therapeutic Chemical code of vaccine	Anatomical Therapeutic Chemical code [10]	NU
Article number of package	Nordic Article Number [11]	NU
Vaccination route	National vaccination route code	NU
Vaccination site	National vaccination site code	NU

NU: not used for identification purposes; THL: National Institute for Health and Welfare.

^a^ Since 2017.

^b^ Since 2017, previously free text.

Each software company designs the data entry into its patient information system software following its own guidelines concerning the use of coding and field validation rules. The process of extracting the data from the patient information systems and submitting them to Avohilmo at THL is fully automated and instructed to be dispatched every night, comprising new primary healthcare visit records and updates to existing primary healthcare visit records each time. The submission pace, i.e. the time interval between the day of submission and the day of vaccination varies between HCCs. In 2015, 84% (129/153) of the HCCs were submitting vaccination records in near real-time, which was defined as a median submission pace of 7 days or less.

## Record linkage

Avohilmo receives the patient information in batches. With regard to vaccinations, these batches contain new and updated records as well as duplicates of old records, i.e. records previously received from another HCC. At THL, the vaccination records (Table 1) are extracted from Avohilmo, pseudonymised and transformed into the NVR with the objectives to identify (i) the administered vaccine and (ii) the vaccination event.

The default variable for identifying the administered vaccine is the lot number, because of its importance in vaccine safety monitoring (Figure 3) and its broad availability regardless of the different patient information systems in use. THL is responsible for the distribution of the vaccines given within the NVP. The list of those vaccines’ lot numbers is continuously updated and incorporated into the NVR for identification purposes. In addition, a contract has been set up with the Finnish Medicines Agency to retrieve bi-annually all lot numbers that are used in Finland but not distributed in the frame of the NVP. All the known lot numbers are used to check against lot numbers that are entered into the patient information systems and submitted to Avohilmo and the NVR by means of exact and approximate string matching, i.e. the comparison of character sequences. Potential spelling mistakes are accounted for by using data cleansing rules and the Levenshtein string similarity metric [16] against the known lot numbers (Table 2).

**Table 2:** Example for the identification of live-attenuated influenza vaccine Fluenz Tetra vaccination records with a single known lot number of FJ2098C using the Levenshtein string similarity metric [16] and a similarity value ≥ 0.7 as an approximate match

Lot number	Cleansed lot number	Similarity value	Trade name	Vaccine identified by
LOT FJ2098C	FJ2098C	(not evaluated)	(not evaluated)	Lot number, exact match
F72098C	F72098C	0.857	(not evaluated)	Lot number, fuzzy match
fj2098.	FJ2098	0.857	(not evaluated)	Lot number, fuzzy match
FJ2O98	FJ2O98	0.714	(not evaluated)	Lot number, fuzzy match
FJ20	FJ20	0.571	Fluenz Tetra	Trade name, exact match
Fluenz Tetra	FLUENZTETRA	0.091	(missing value)	(not identified)

The default variable for identifying the administered vaccine is the lot number, cleansed for potential spelling mistakes. If this identification process fails, the trade name is evaluated. Having valuable information, e.g. the trade name, entered in the wrong field and other fields, e.g. the actual field for the trade name, empty or also with the wrong kind of information, can make the vaccine identification as part of the record linkage impossible.

Matches over a certain level (data-driven set to 0.7) that unambiguously match against the known lot numbers of one vaccine are interpreted as successfully identified. If this identification process fails, the record of the trade name is used and evaluated by exact string matching including common spelling variations.

Vaccination events are defined as unique combinations of three key variables: (i) personal identity code, (ii) identified administered vaccine, and (iii) date of vaccination. The record describing the vaccination event that was received first is kept for further processing and analysis.

## Alignment with commercial patient information systems

In addition to establishing nationwide coding standards, collaboration with the patient information systems’ software companies is pivotal, since they are responsible for designing the data retrieval based on their patient information systems, and for creating the data format for data transfers. In spring 2010, the collaboration was initiated by reviewing the data entry in each of the patient information systems. In autumn 2010, THL piloted a retrospective data collection of vaccination records as part of a planned nationwide collection of pandemic influenza vaccination records via the patient information systems’ software companies. This data collection was also used by THL to assess the availability, completeness, and quality of the available information. Subsequently, three additional retrospective data collections have been conducted in 2011, 2014, and 2015, respectively, covering all vaccinations, but limited to certain HCCs and time periods. By incorporating the retrospective data collections through the same record linkage steps that are applied to the Avohilmo data, the NVR currently covers nationwide records of vaccinations given in Finnish HCCs since 2009 (Figure 1).

## Education and centralised guidance for healthcare professionals

Guidelines for recording vaccinations are available online [17]. The recording of lot numbers is emphasised because of the lot level traceability requirements. When the majority of HCCs started submitting real-time data, in spring 2012, a two-day educational workshop was held for HCCs’ staff responsible for the guidance of the patient information recording, as well as representatives of the patient information systems’ software companies (Figure 1). The purpose of the workshop was to alert the healthcare providers about the creation of the NVR, to standardise recording conventions, and to learn about the practical challenges in everyday work when it comes to computer-based recording.

Currently, for any feedback from the HCCs or staff involved in the patient information systems, a NVR contact person can be reached via phone and email. Thus, feedback can be evaluated promptly and questions may even be answered immediately. Feedback is also exchanged during four to 12 annual field visits in the HCCs by teams composed of experts from the NVR, Avohilmo, and THL’s Vaccination Programme Unit. These visits are used for both education and guidance of healthcare professionals and investigation of deficiencies in data quality. Often including also representatives of the software companies, these joint dialogues have led to HCC-specific modifications in the recording conventions, as well as to software interface changes that have made the recording of vaccinations easier, more complete, and less error-prone.

Moreover, THL organises monthly web-based seminars for those working with vaccinations. Depending on the topic, reminders on the need for accurate data entry and the different ways of using the data are covered during these seminars.

## Data quality assessment

From the start of the NVR, completeness of vaccination data has been investigated in order to assess deficiencies in data entry and data collection. Nowadays, the completeness of vaccination data is routinely monitored every month for each HCC [18] (i) for the population as a whole to control whether the HCC has been submitting vaccination records at all and (ii) for children younger than 2 years, who are recommended to follow a tight vaccination schedule of 10 or more vaccinations in the first 2 years of life [5]. For the whole population, the ratio of the monthly number of vaccination events other than seasonal influenza vaccination and the yearly count of residents living in the municipalities served by the HCC is calculated. For children younger than 2 years, the ratio of the monthly number of diphtheria, tetanus, acellular pertussis, inactivated polio, and *Haemophilus influenzae* type b (five-in-one) and measles, mumps, and rubella (three-in-one) vaccination visits and the number of children younger than 2 years living in the municipalities served by the HCC is calculated. The rolling 6-month average of these ratios is compared with a fixed cut-off, one for each ratio [18]. The choice of the two cut-offs is data driven and aimed at identifying notable temporal changes in the data flow from the HCCs. These changes are assumed to be caused, among others, by modifications in software programmes, recording conventions, or data submission.

Additionally, an alert system has been deployed that reports monthly unexpected changes in the HCCs’ reporting behaviour based on those ratios. Taking into account each HCC’s median submission pace of the last 30 days, it checks the data completeness of the last month and produces a list of HCCs with an insufficient amount of documented vaccination events. This system is devised to react early to systematic data entry and data dispatch problems. The development of the alert system has largely been guided through experience.

In autumn 2012, after an update to one patient information system software, all vaccination records of newly and recently born children were accidentally omitted from real-time data submissions in the HCCs using that particular software. At this time, only one general indicator for vaccination data completeness similar to the first one described above (i) was available, but not regularly checked. Since records were still accumulating for the rest of the population served by these HCCs, it took several months to detect this deficiency, which led to the implementation of a more elaborate alert system including the indicator for childhood vaccination data completeness (ii) and automated, monthly alert reports. In spring 2014, a similar situation occurred affecting adolescent human papillomavirus (HPV) vaccinations after an update to another patient information system software. This time, the alert system was able to point out the anomaly in less than two months. After both incidents, the respective software companies were contacted and retrospective data collections were conducted in order to fill the identified NVR’s gaps.

## Reporting

Starting with autumn 2013, THL has been reporting annual nationwide and HCC-specific vaccination coverage figures of the Finnish childhood vaccination programme, adolescent HPV vaccination programme and seasonal influenza vaccination programme [6] (Figure 1). These are also available in a form of interactive maps, providing user-friendly access to aggregated nationwide vaccination data. Only HCCs meeting the criterion for data completeness for all the months covered by the observation period of interest, e.g. from birth until the age of 2 years in the case of early-childhood vaccinations, are included in nationwide reports, which are online available [19]. The same quality control process is applied for any vaccine impact analysis using the NVR: in a recent effectiveness study of the live attenuated and the inactivated influenza vaccine in 2-year-olds, the individuals covered by the HCCs that did not meet the data completeness criterion, i.e. 5% of the study population, were omitted from the population-based analysis [20].

Data on the absolute count of vaccinations are included in the reports as well, allowing a direct comparison with the HCCs’ own records. In addition, summaries of the vaccine identification process (Table 2) are shared with the HCCs. After continuous efforts emphasising the importance of the lot number, both through education and by means of periodical reports, the quality of these data entries has improved from 94% (1,570,169/1,674,905) identified by lot number in 2012 to 97% (2,063,669/2,121,646) in 2015 and from 1% (21,675/1,674,905) unidentified in 2012 to 0% (6,257/2,121,646) in 2015.

Moreover, the amount of incoming vaccination data is monitored weekly and internal online follow-ups provide near real-time vaccination coverage information for all vaccines and age groups.

## Challenges and future perspectives

The constantly reshaping administrative areas (Figure 2) and the variety of patient information system software programmes and their versions have made the establishment of the NVR in Finland particularly challenging. The current mechanisms constantly required for maintaining the NVR are (i) methods for record linkage, (ii) education of healthcare workers, (iii) data quality assessment, and (iv) continuous reporting.

The vaccine lot number is used as the key variable for identifying the vaccine. From the coding point-of-view, the lot number is a suboptimal choice for vaccine identification, because it is not feasible to develop a pre-coded data entry for the lot number in the patient information systems. Instead, free text is required. A potential improvement would be to use barcode readers, but unfortunately vaccine manufacturers do currently not include the lot number in the vaccines’ barcodes in Europe. However, with the listed four quality control mechanisms in place, the quality of computer-based recording of NVP vaccinations in Finland has improved to a degree that almost all vaccines are currently identified on a lot level [18].

When collecting a large amount of real-time data in an automated fashion, and from several data providers and software systems, special attention is required to detect any anomalies in data. Any unexpected changes in the process of data entry and data dispatch can lead to inadvertent omission of records, as exemplified by the problems experienced during the first years of the NVR. In order to detect these changes, an alert system has been deployed. However, since it is not possible to anticipate all possible anomalies, developing and maintaining the alert system based on previous experience, is a continuous process.

Maintaining the NVR also requires continuous interaction and dialogue with the HCCs. A pivotal tool for such interaction is permanent provision of reports that summarise vaccination records at HCC-level. Due to regional and temporal gaps in the completeness of the data, only periodical (annual) reporting has been employed so far. However, the plan is to gradually move towards continuous reporting. Continuous reporting was piloted during the influenza season 2015/16, when the accumulation of influenza vaccinations documented in the NVR was reported weekly on a public THL webpage [21], and is continued in 2016/17 [22].

At the moment, the NVR contains vaccinations given within the public primary healthcare system. However, Avohilmo is currently being expanded also to the private primary healthcare. Furthermore, other national registers like the Hospital Discharge Register and the Medical Birth register are being prepared to include also vaccination information into their data content and collection. The purpose of these additions is to include also vaccinations given in hospitals and at birth clinics into the NVR.

In parallel with the efforts to add the collection of vaccination records into other national registers, a project has been initiated, which explores the possibility to utilise the recently established Finnish National Patient Archive (Kanta) [23]. Kanta is a nationwide system developed for nationwide access to patient information for both healthcare workers and citizens themselves. Through Kanta, citizens can browse personal vaccination records via an online server. Parallel efforts to expand data collections and utilise already collected data for register purposes is especially topical now, when Finland is introducing a health, social services and regional government reform, one of the biggest ever administrative and operational overhauls in Finland [24].

## Conclusion

Constant monitoring of the quality of a population-based vaccination register is a prerequisite for a continuous and reliable evaluation of both the vaccine uptake and impact on a national level. The presented quality control measures developed to monitor the validity of NVR data in Finland have proven useful to improve the quality and completeness of the register. Thus, this process documentation may serve as a basis to improve the design and the quality of other vaccination or healthcare registers.
